# *SERPINB2*, an Early Responsive Gene to Epigallocatechin Gallate, Inhibits Migration and Promotes Apoptosis in Esophageal Cancer Cells

**DOI:** 10.3390/cells11233852

**Published:** 2022-11-30

**Authors:** Zikai Chen, Yifu Wei, Yuzhong Zheng, Hui Zhu, Qian Teng, Xianglan Lin, Fengnian Wu, Fei Zhou

**Affiliations:** Department of Cell Biology, Hanshan Normal University, Chaozhou 521041, China

**Keywords:** esophageal cancer, EGCG, *SERPINB2*, apoptosis

## Abstract

Esophageal cancer is a lethal disease that frequently occurs in developing countries, the incidence of which could be declined by drinking EGCG-enriched drinks or food. *SERPINB2*, whose complex functions and regulations are not yet fully understood, are induced by multiple inflammatory molecules and anti-tumor agents. Here, we identify 2444 EGCG-regulated genes in esophageal cancer cells, including *SERPINB2*. EGCG treatment recruits NF-κB at the promoter and enhancers of *SERPINB2* and activates gene transcription, which is repressed by NF-κB knockdown or inhibition. Loss of *SERPINB2* leads to a faster migration rate and less expression of Caspase-3 in cancer cells. Our study demonstrates that *SERPINB2* is a new tumor-suppressor gene involved in cell movement and apoptosis and could be a therapeutic target for esophageal cancer.

## 1. Introduction

Esophageal cancer is a common digestive-tract cancer, with approximately 604,000 new cases and 544,000 deaths worldwide in 2020. The incidence rate is high in developing countries in Eastern Asia and Southern Africa [1]. Progress has been made in diagnosis and treatment, but the overall curative effect and prognosis of esophageal cancer are still poor [2], so it is urgent to discover novel therapeutic agents and targets.

Epigallocatechin gallate (EGCG), a natural dietary polyphenol compound with low toxicity, can not only energize gastrointestinal digestion, reduce blood lipids, and boost the immune system, but also functions as an inhibitor of bacteria, and has significant preventive and therapeutic effects on esophageal cancer [3,4]. However, the mechanism of EGCG as an anti-cancer agent is not completely clear, partly due to its multiple molecular targets [3].

Serine Proteinase Inhibitor 2 (*SERPINB2*), also named Plasminogen Activator Inhibitor 2, is one of the most significantly up-regulated genes during cellular stress. There are two forms of SERPINB2: a 47-kD intracellular form without glycosylation, and a secreted 60-kD glycosylated form [5], but they are functionally similar [6]. Early studies found that SERPINB2 could bind to and inhibit urokinase and tissue plasminogen activators (uPA and tPA) [7]. *SERPINB2* is involved in a variety of biological processes, such as reducing bleeding times by affecting platelet aggregation [8], protecting mice kidneys from damage and fibrosis [9], impairing osteoblastic differentiation of bone marrow stromal stem cells [10], and acting as a biomarker for asthma [11]. In addition, the expression of *SERPINB2* increased when cells underwent senescence. *SERPINB2* was a direct target of p53, which was conversely up-regulated by SERPINB2 [12]. It was highly inducible under inflammatory conditions, and its dysregulated expression may cause immune-system-related diseases [13,14].

More importantly, *SERPINB2* was directly related to tumor promotion and poor prognosis in various cancers such as bladder [15], colorectal [16], endometrial [17], and ovarian [18] cancers. *SERPINB2* might act as a regulator or biomarker for predicting metastatic progression in breast and liver cancers [5,19]. *SERPINB2* expression significantly increased in response to various tumorigenic agents in multiple cancer stem cell types [20]. However, the function of *SERPINB2* has not been characterized in esophageal cancer. Herein we identified *SERPINB2* as an EGCG-regulated tumor-suppressor gene that was mediated by NF-κB and confirmed the role of *SERPINB2* in cell migration and apoptosis.

## 2. Material and Methods

### 2.1. Cell Culture

Human esophageal squamous cell carcinoma (ESCC) cell lines KYSE150 and KYSE510 were generously provided by Dr. Xu Liyan at Shantou University Medical College. The cells previously described [21,22] were maintained at 37 °C with 5% CO_2_ in RPMI1640 medium (Biosharp Life sciences, Beijing, China) supplemented with 10% FBS (TransGen, Beijing, China). For EGCG stimulation assays, we inoculated the cells at a concentration of 1.6 × 10^5^/mL in full-serum medium and changed to RPMI1640 medium without FBS 24 h later, then treated the cells with EGCG at indicated concentrations. We also performed EGCG stimulation without changing the medium. Inhibition of p65 was performed by treating the cells with 5 μM BAY11-7082 (Beyotime Biotechnology, SF0011, Shanghai, China) for 1~3 h. 

### 2.2. Gene Knockdown, Gene Overexpression, and Cell Proliferation Assay

For EGCG concentration-series and time-series assay, KYSE150 and KYSE510 cells were treated with 0–100 μM EGCG (Guangdong Yilong, Chaozhou, China) for 24 h or 60 μM EGCG for 0–60 h with or without FBS. For the *SERPINB2* knockdown assay, two antisense oligonucleotides (ASOs, Accurate Biotechnology, Hunan, China) targeting *SERPINB2* mRNA were used to transfect the cells. The sequences and design of ASOs are shown in Table S1. For p65 knockdown, SignalSilence^®^ NF-κB p65 siRNA II #6534 (Cell Signaling Technology, Shanghai, China) targeting RELA mRNA was used to transfect the cells [23]. Briefly, 100 µL cells (4 × 10^4^) were inoculated in a 96-well plate one day before transfection. An amount of 0.1 µL of 10 µM ASO or siRNA was added to 10 µL Opti-MEM (Gibco, Thermo Fisher Scientific, Shanghai, China) and mixed with 0.3 µL of Lipofectamine RNAiMAX reagent (Invitrogen, Shanghai, China). The mixture was incubated at room temperature for 5 min and dripped into the wells. The cells were then cultured for 24 h in the 37 ℃ incubator. *SERPINB2* overexpression was conducted using the plasmid EX-Z6805-M98 (GeneCopoeia, Guangzhou, China) and Lipo8000^TM^ (Beyotime Biotechnology, C0533) according to the manufacturer’s instructions. The vector pEZ-M98 was used as control.

The supernatant was removed and replaced with RPMI1640 medium containing 10% cell counting kit 8 (CCK8) solution (MCE, Shanghai, China). The cells were incubated for an additional hour and measured at 450 nm with a MULTISKAN MK3 spectrophotometer (Thermo Scientific, Shanghai, China).

### 2.3. RNA Extraction and qRT-PCR

Total RNA was isolated using Total RNA Isolation Reagent (Biosharp Life sciences, Beijing, China) according to the manufacturer’s instructions. Reverse transcription was performed using Hifair II 1st Strand cDNA Synthesis SuperMix with a gDNA digester (Yeasen, HB181210, Shanghai, China). Quantitative real-time PCR was performed using SYBR Green Master Mix (Yeasen, HB181203, Shanghai, China) on a Lightcycler Real-Time PCR System (Roche, Beijing, China). The relative expression level of the target genes and the relative fold change were normalized to GAPDH and the control, respectively. The sequences of primers (Tianyihuiyuan, Guangzhou, China) are shown in Table S2.

### 2.4. RNA-seq and Screening for Differentially Expressed Genes (DEGs)

Quality control was carried out for RNA samples extracted as described in 2.3 (Table S3). Then, MGISEQ-2000 RNA-Seq was performed by Beijing Genomics Institute (BGI). We filtered out DEGs in two steps: first, the genes had a fold change greater than 2 (or smaller than 0.5) and a false discovery rate lower than 0.05. Second, the average FPKM of three replicates of the selected genes was more than 1.0 for either control or EGCG-treated samples. The analysis for RNA-seq data was conducted using Dr. Tom System developed by BGI (https://biosys.bgi.com (accessed on 5 January 2021)).

### 2.5. Western Blotting

Cells were directly lysed using a home-made loading buffer (12 mM Tris-HCl (pH6.8), 5% glycerol, 0.4% SDS, 2.88 mM β-mercaptoethanol, 0.02% bromophenol blue). Protein extracts were boiled for 10 min and then separated on SDS–PAGE gels. Protein amounts were adjusted according to the housekeeping gene GAPDH. 

Antibodies used for Western blots were as follows: SERPINB2 rabbit polyclonal antibody (ABclonal, A15297, Wuhan, China), GAPDH mouse monoclonal antibody (Beyotime Biotechnology, AF0006), Caspase-3 rabbit polyclonal antibody (Beyotime Biotechnology, AF0081). Secondary antibodies were HRP-conjugated goat anti-mouse IgG (Beyotime Biotechnology, A0216) and peroxidase-conjugated goat anti-rabbit IgG (Beyotime Biotechnology, A0208).

### 2.6. Chromatin Immunoprecipitation (ChIP)

ChIP assays were performed using a Chromatin Immunoprecipitation Kit (Millipore, 17-611, Guangzhou, China) as previously described [24]. Cells were inoculated in 10 cm dishes, treated with EGCG for 2 h, and fixed with 4% formaldehyde (Sigma, 252549, Shanghai, China). After DNA fragmentation, 2–3 µg ChIP-grade antibodies against anti-IgG (Beyotime Biotechnology, A7016) and anti-p65 (Abcam, ab16502, Shanghai, China) were used to perform ChIP assays with protein A/G magnetic beads (MCE, Shanghai, China). Immunoprecipitated DNA was purified using a DNA purification kit (Axygen, Corning Life Sciences, Wujiang, China) and applied to qPCR. The results were normalized to the input DNA. The sequences of the primers are listed in Table S4.

### 2.7. Cell Migration Assay

Cell migration was determined using a scratching assay. Cells were inoculated in a 96-well plate and grew to 100% confluency. A scratch was generated by using a sterilized 10 μL pipette tip. The cells were washed twice with PBS and cultured in RPMI1640 medium in the absence of FBS, followed by ASO-mediated *SERPINB2* knockdown, with or without 60 μM EGCG treatment. Images were captured at different time points using a microscope (Olympus IX73 or Nikon Eclipse TS100).

### 2.8. Statistical Analysis

A paired-sample T-test was used to determine if the difference was significant between the two groups of data (*p* < 0.05). One-way ANOVA analysis was employed for more than two samples. All data were calculated with at least three independent experiments and shown as the mean ± standard deviation (SD).

## 3. Results

### 3.1. EGCG Inhibits Esophageal Cancer Cell Proliferation in a Dose- and Time-Dependent Manner

**Figure 1:**
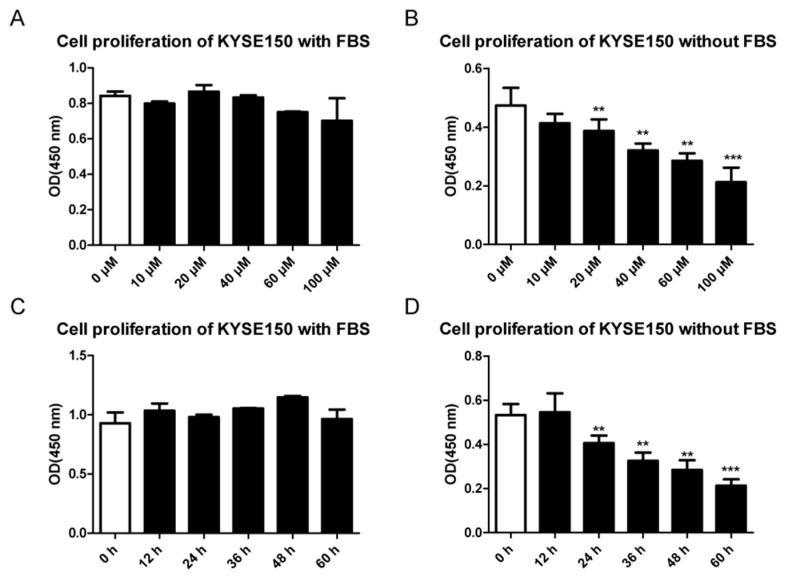
Cell proliferation of KYSE150 under different conditions. (**A**,**B**) Cells were treated with EGCG at concentrations from 0~100 μM with (**A**) or without (**B**) FBS for 24 h. (**C**,**D**) Cells were treated with 60 μM EGCG at a series of time points from 0~60 h with (**A**) or without (**B**) FBS. Data are shown as mean ± SD. *n* = 3, **: *p* < 0.01; ***: *p* < 0.001.

To test the efficacy of EGCG in cancer cell growth, we treated KYSE150 and KYSE510 cells with EGCG at a series of concentrations and time points with or without FBS. With the presence of FBS, cancer cell proliferation was marginally affected at a concentration of up to 100 μM for 24 h or at 60 μM up to 60 h (Figure 1A,C and Figure S1A,C). However, in the absence of FBS, both cell lines showed significantly lower growth rates when treated with a generally low concentration of EGCG. The half maximal inhibitory concentration (IC50) was approximately 60 μM without FBS, but it was approximately 240 μM with the presence of FBS (Figure 1B and Figure S1B,E). In addition, the inhibitory effect of EGCG on cell proliferation was also treatment-time-dependent (Figure 1D and Figure S1D). To examine the molecular functions of EGCG with minimum influence on cell viability, we adopted 60 μM of EGCG without FBS for the following assays. 

### 3.2. mRNA Sequencing Reveals Early Responsive Genes of EGCG in Esophageal Cancer

**Figure 2:**
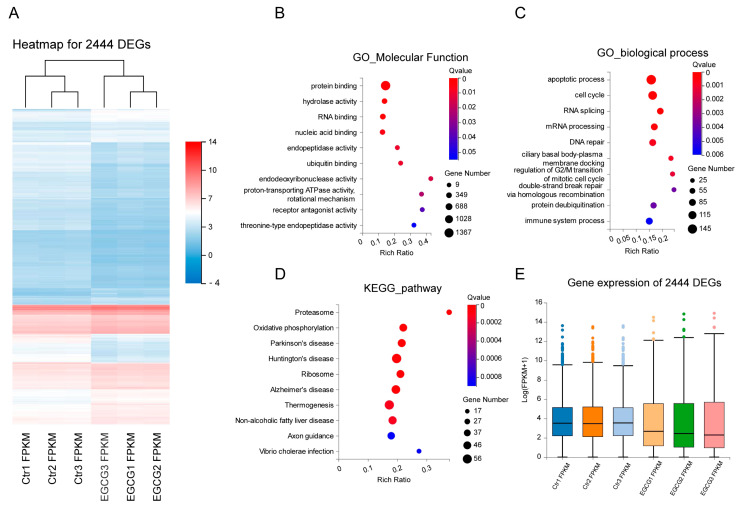
Analysis of 2440 differentially expressed genes (DEGs). (**A**) Heatmap showing the expression patterns before and after EGCG treatment. (**B**) GO analysis of molecular function for DEGs. (**C**) GO analysis of the biological process for DEGs. (**D**) KEGG pathway enrichment for DEGs. (**E**) The expression level of DEGs before and after EGCG treatment.

To systematically characterize EGCG-regulated genes at the early stage, we treated K150 cells with 60 μM of EGCG for 6 h and extracted mRNA for sequencing. We identified 2440 differentially expressed genes (DEGs) by comparing the samples treated with and without EGCG. A total of 1072 genes were up-regulated while 1372 genes were down-regulated (Figure 2A and Figure S2B). In order to explore the function of EGCG on esophageal cancer cells, we further performed enrichment of gene ontology and KEGG pathways for the DEGs. For molecular function, the DEGs were highly enriched in hydrolase and endopeptidase activity (Figure 2B). For the biological process, they were enriched in the apoptotic process and DNA repair (Figure 2C). For KEGG pathways, the EGCG-regulated genes were also enriched in Proteasome (Figure 2D), indicating active protein degradation upon EGCG treatment. There was no significant difference in the global expression of all detected genes between the control and the EGCG-treated samples (Figure S2A). On the contrary, the median expression levels of the DEGs were lower than that of the control groups (Figure 2E), indicating a general suppressive effect of EGCG on the expression of regulated genes.

### 3.3. Validation of EGCG-Regulated Genes by qRT-PCR

**Figure 3:**
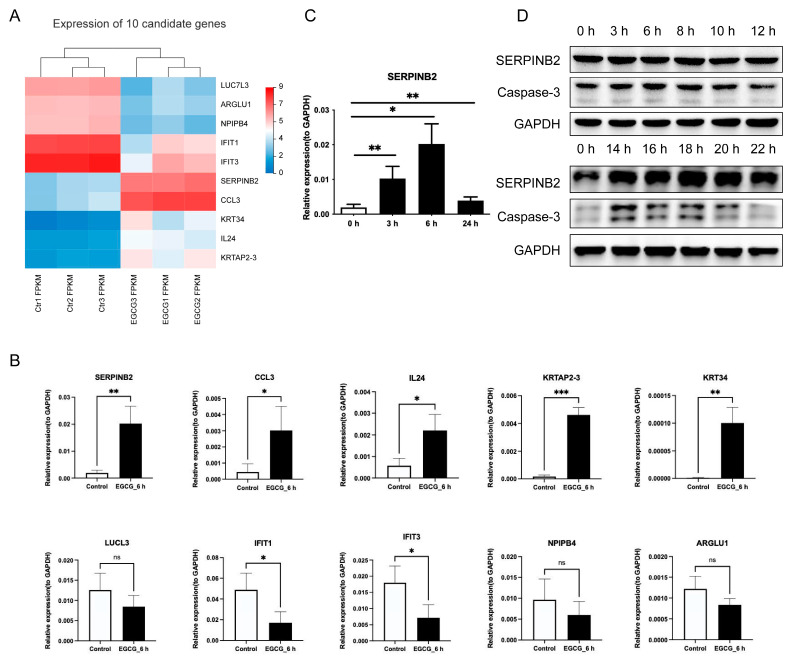
Validation of DEGs in KYSE150 cells. (**A**) Heatmap showing the expression levels of the top 10 up-regulated and down-regulated genes in RNA-seq. (**B**) Verification of DEGs after EGCG treatment for 6 h in KYSE150 cells. (**C**) Relative expression of SERPINB2 after EGCG treatment at 0, 3, 6, and 24 h in KYSE150 cells. (**D**) Changes of SERPINB2 and Caspase-3 protein levels at different time points in KYSE150. Data are shown as mean ± SD. *n* = 3, *: *p* < 0.05; **: *p* < 0.01; ***: *p* < 0.001, ns: not significant.

To verify the DEGs identified by RNA sequencing, we selected 5 up-regulated genes (*SERPINB2*, *CCL3*, *IL24*, *KRTAP2-3*, and *KRT34*) and 5 down-regulated genes (*LUCL3*, *IFIT1*, *IFIT3*, *NPIPB4*, and *ARGLU1*) and performed qRT-PCR to determine the expression levels of these genes before and after EGCG treatment for 6 h. The trends for the change of all the ten genes were consistent with the sequencing data (Figure 3A,B and Figure S3A), though the differences were not significant for *LUCL3*, *NPIPB4*, and *ARGLU1* in KYSE150 and for *ARGLU1* in KYSE510 (Figure 3B and Figure S3A). *SERPINB2* was the most up-regulated gene (16-fold higher than the control) among 2440 DEGs. Therefore, we further explored the expression trend in *SERPINB2* at different time points. *SERPINB2* was highly induced by EGCG at the early time points and reduced after 24 h, yet still significantly higher than the control (Figure 3C and Figure S3B). The protein level of SERPINB2 did not change before 12 h and increased from 14 h to 22 hour’s treatment in KYSE150. On the other hand, the elevation of SERPINB2 in KYSE510 was as early as 3 h. Intriguingly, we found synchronous fluctuation of Caspase-3 and SERPINB2 during the time course assay (Figure 3D and Figure S3C).

### 3.4. SERPINB2 Negatively Regulates Cell Migration and Enhances Apoptosis in Esophageal Cancer

**Figure 4:**
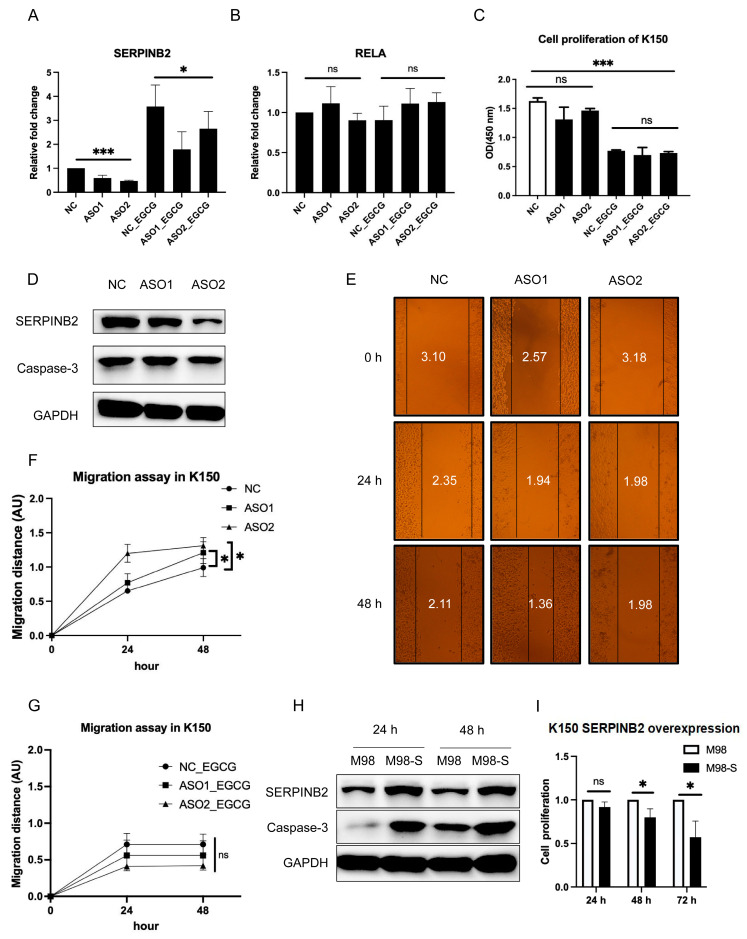
SERPINB2 is associated with cell migration and apoptosis in KYSE150. A-B. Relative fold change of SERPINB2 (**A**) and RELA (**B**) 24 h after knockdown with antisense oligonucleotide (ASO) targeting SERPINB2. (**C**) Cell viability after ASO-mediated knockdown of SERPINB2. (**D**) Western blotting showing down-regulation of SERPINB2 and Caspase-3 after SERPINB2 knockdown. (**E**) Wound healing assay showing cell migration after SERPINB2 knockdown without EGCG treatment. (**F**,**G**) Statistical analysis for cell migration distance of three independent assays without (**F**) or with (**G**) EGCG. (**H**) Western blotting showing up-regulation of SERPINB2 and Caspase-3 after SERPINB2 overexpression. (**I**) Cell viability determined by CCK8 assay after SERPINB2 overexpression. Data are shown as mean ± SD. *n* = 3, *: *p* < 0.05; ***: *p* < 0.001, ns: not significant.

To investigate the function of *SERPINB2* in esophageal cancer cells, we performed knockdown experiments using antisense oligonucleotides (ASO) (Figure 4A,D and Figure S4A,D). The expression of *SERPINB2* significantly reduced after gene knockdown regardless of EGCG treatment, and the *SERPINB2* levels were constantly higher in the presence of EGCG versus no EGCG. (Figure 4A and Figure S4A). Unexpectedly, knockdown of *SERPINB2* did not affect cell viability regardless of EGCG treatment (Figure 4C and Figure S4C). Importantly, we observed a significant increase in cell migration of KYSE150 (Figure 4E,F) and KYSE510 (Figure S4E,G) in the absence of EGCG after *SERPINB2* knockdown. However, the migration-promoting effect of *SERPINB2* downregulation was prohibited by EGCG treatment (Figure 4G and Figure S4F,H). In addition, cells treated with *SERPINB2*-ASO showed decreased protein levels of Caspase-3 (Figure 4D and Figure S4D), suggesting that *SERPINB2* was closely related to cell apoptosis. Consistently, overexpression of *SERPINB2* induced high expression of Caspase-3 and impaired cell viability (Figure 4H,I and Figure S4I).

### 3.5. EGCG Regulates the Expression of SERPINB2 via Enrichment of NF-κB at Its Promoter and Enhancers

**Figure 5:**
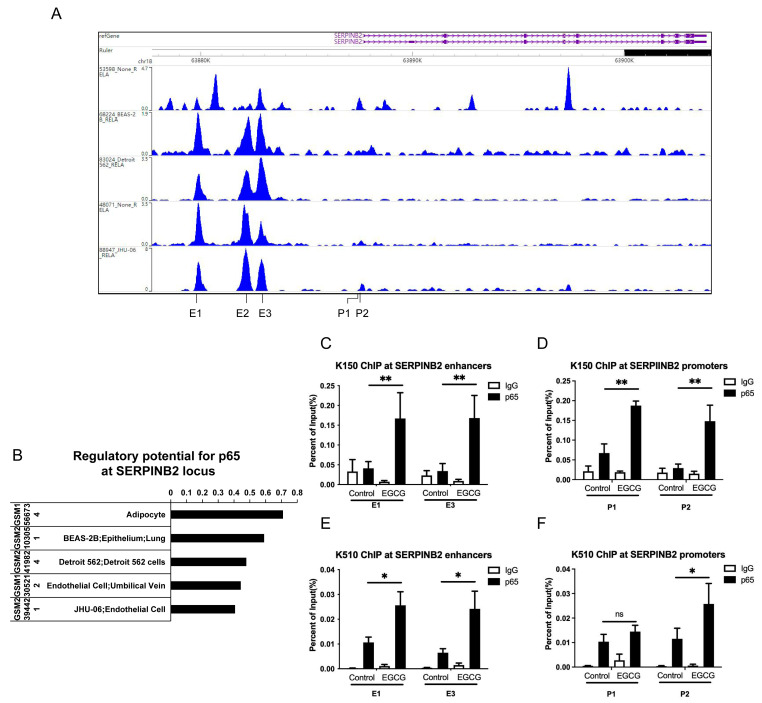
Prediction and verification of p65 binding sites on the promoter and enhancers of SERPINB2. (**A**) Cistrome screenshot showing p65 binding peaks near the SERPINB2 locus in adipocyte, lung epithelial cells, Detroit cells, and endothelial cells. (**B**) Regulatory potential for p65 at SERPINB2 locus within 5 cell lines. (**C**,**D**) ChIP-qPCR in KYSE150 cells before and after EGCG treatment showing p65 enrichment over the enhancer E1 and E3 (**C**) and the promoter (**D**) regions (**E**,**F**). ChIP-qPCR in KYSE510 cells before and after EGCG treatment showing p65 enrichment over the enhancer E1 and E3 (**E**) and the promoter (**F**) regions. Data are shown as mean ± SD. *n* = 3, *: *p* < 0.05; **: *p* < 0.01; ns: not significant.

Next, we aimed to explore the regulatory mechanism of EGCG on *SERPINB2* expression. To this end, we searched for potential transcriptional factors (TFs) that bind to the promoter and enhancer regions of *SERPINB2* on the Cistrome Browser [25]. Currently, no ChIP-seq data are available for esophagus cancer cells, so we screened the TFs using five independent data sets (GEO accession ID: GSM1566734, GSM2103051, GSM2419824, GSM1305212, GSM2394421), including adipocyte, lung epithelium cells, Detroit 562 cells, and endothelium cells (Figure 5A). We identified the subunit of NF-κB, p65 with a high capacity of binding and regulatory potential at both regions (Figure 5B). Thus, we conducted ChIP PCR to verify three p65 binding sites at the enhancer region and two at the promoter before and after EGCG treatment in both KYSE150 and KYSE510. We observed significant increases for p65 enrichment at the two sites of the promoter, as well as the enhancer E1 and E3 after EGCG treatment for 2 h (Figure 5C–F). However, p65 binding at E2 was not affected by EGCG treatment (Figure S5A,B).

### 3.6. Knockdown of NF-κB Down-Regulates SERPINB2 Expression and Inhibits Cell Death

**Figure 6:**
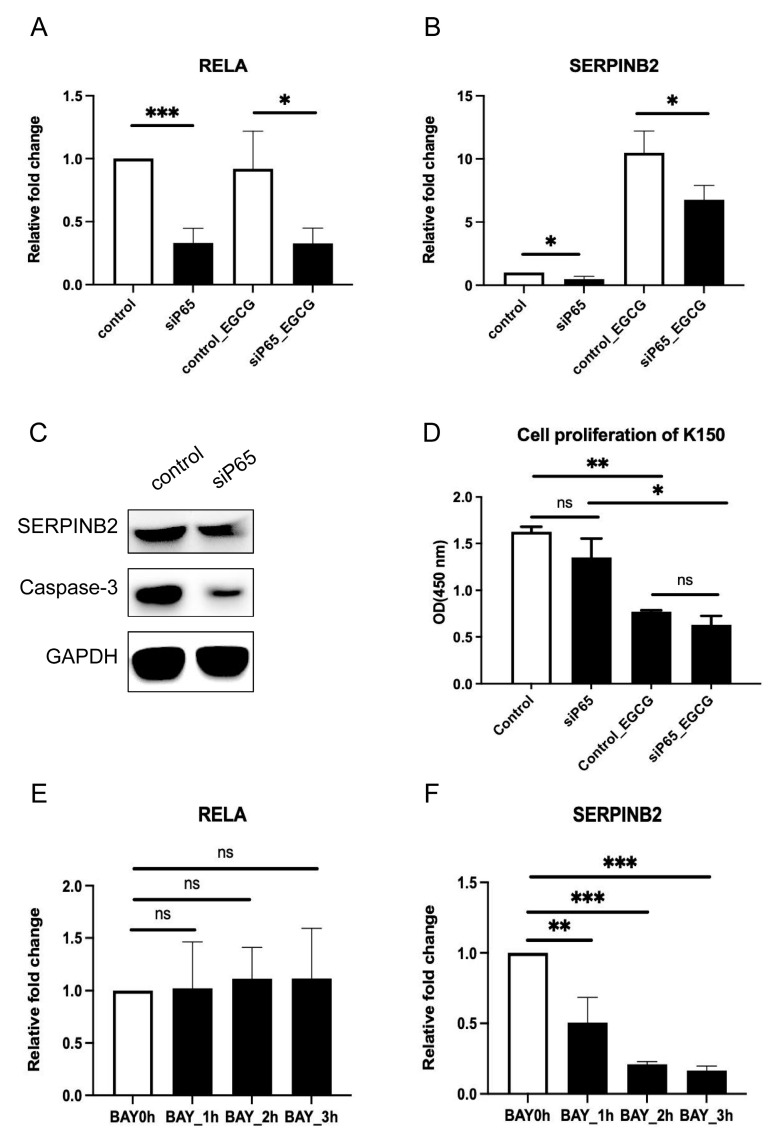
p65 regulates the expression of SERPINB2 in KYSE150 cells. (**A**,**B**) Relative fold change of RELA (**A**) and SERPINB2 (**B**) after p65 knockdown with or without EGCG. (**C**) Western blotting showing protein levels of SERPINB2 and Caspase-3 after p65 knockdown. (**D**) Cell proliferation after p65 knockdown. (**E**,**F**) Relative fold change of RELA (**E**) and SERPINB2 (**F**) expression after p65 inhibition. Data are shown as mean ± SD. *n* = 3, *: *p* < 0.05; **: *p* < 0.01; ***: *p* < 0.001, ns: not significant.

To further confirm the function of NF-κB in regulating *SERPINB2*, we performed p65 knockdown and NF-κB inhibition in both KYSE150 and KYSE510. siRNA-mediated knockdown of p65 significantly down-regulated the expression of *SERPINB2* in both cell lines, even under the condition of EGCG treatment (Figure 6A–C and Figure S6A–C). In addition, p65 knockdown also caused inhibition of cell apoptosis in KYSE150 (Figure 6C) but not in KYSE510 (Figure S6C). However, p65 knockdown did not affect cell viability, which was consistent with the effect of *SERPINB2* knockdown (Figure 4C, Figure 6D, Figures S4C and S6D). Similarly, BAY-11-7082-mediated NF-κB inhibition significantly decreased the expression of *SERPINB2* (Figure 6F and Figure S6F), regardless of the influence on RELA expression (Figure 6E and Figure S6E). These results indicate that NF-κB is upstream of SERPINB2 but not the other way around (Figure 4B and Figure S4B).

## 4. Discussion

EGCG, the most abundant catechin in green tea, has been shown to suppress tumor development in cancer but with low bioavailability [3,26]. In this study, we showed that EGCG was more potent in the medium without FBS than that with FBS, indicating that some ingredients in the serum may decrease the efficacy of EGCG, possibly by mediating its degradation or inhibiting its cell import. This result may suggest that esophageal cancer cells are more sensitive to anti-tumor agents under “starvation” conditions. We depicted the change of transcriptional landscape after EGCG stimulation in esophageal cancer cells and revealed its effects on cellular protein degradation and apoptosis. This study also provides more than 2000 potential EGCG-regulated genes for future study.

**Figure 7:**
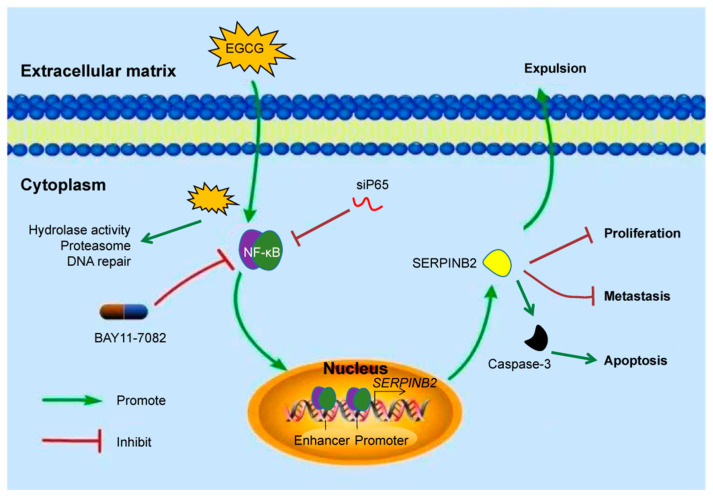
Diagrammatic sketch showing the regulations of EGCG on *SERPINB2* expression and related pathways.

EGCG treatment induced *SERPINB2*, which impaired cell growth and metastasis since *SERPINB2* knockdown enhanced cell migration and *SERPINB2* overexpression promoted cell death. These findings suggest that EGCG exerts its effect partly by upregulating *SERPINB2*. The roles of SERPINB2 are contentious and seemingly contradictory under different circumstances, as cancer-promoting and -suppressing functions were reported [27,28], even within the same cancer type [29,30]. SERPINB2 overexpression inhibited invasiveness and metastasis in liver cancer and pancreatic cancer [29,31]. On the contrary, SERPINB2 expression induced cancer cell migration and was associated with a poor survival rate in cholangiocarcinoma patients [32]. In this study, we demonstrated that SERPINB2 was a tumor suppressor which inhibited metastasis and induced apoptosis (Figure 7), suggesting that it could be a novel therapeutic target for treating esophageal cancer.

Previous studies have shown that the extraordinary *SERPINB2* induction in cells is overly higher than that required to inhibit any protease [33], indicating that the function of *SERPINB2* was for storage or transport [34]. The protein level of SERPINB2 was accumulated when the cells were treated with EGCG within 24 h, but the increase was not comparable to the RNA elevation. This phenomenon was likely due to the short half-life of *SERPINB2* protein and clearance or expulsion by cancer cells.

External stimuli often cause variations in the binding affinity of related transcription factors [24]. The epigenetic regulation of *SERPINB2* has been explored but not fully understood [34]. For example, the expression of *SERPINB2* was coordinated by the recruitment or departure of the pause-releasing kinase P-TEFb and the pause-inducing protein NELF at the promoter region [35]. In addition, *SERPINB2* transcription was controlled by the interaction of a silencer and distal transactivator region upstream of the transcription start site [36]. The aryl hydrocarbon receptor was involved in mediating the expression of *SERPINB2* indirectly by regulating its enhancer RNA [37,38]. Conversely, the occupancy of the GATA-type transcription factor Trps1 on the regulatory region of *SERPINB2* repressed its expression [39,40]. Our study revealed that transcription factor NF-κB was recruited to the promoter and enhancers of *SERPINB2* and involved in the transcriptional activation of *SERPINB2* (Figure 7). Earlier studies showed that *SERPINB2* could be induced by TNF-α [5], possibly through the same pathway, which requires further investigation.

The current study has some limitations. First, both cell lines derived from ESCC and cells from other types of cancer, such as adenocarcinoma, can be recruited to support our conclusions. Second, although the artificial culture condition without FBS reduced experimental disturbance, it could not mimic the situation in the humoral system. Last but not least, apoptosis is a complicated process involving multiple proteins and has not been thoroughly evaluated in this study.

## Data Availability

The differentially expressed gene list generated by RNA-seq can be found in Table S5.

## Supplementary Materials

The following supporting information can be downloaded at: https://www.mdpi.com/article/10.3390/cells11233852/s1, Figure S1: Cell proliferation of KYSE510 under different conditions; Figure S2: Global analysis for RNA-seq of KYSE150 cells; Figure S3: RT-qPCR to verify DEGs in KYSE510 cells; Figure S4: SERPINB2 is associated with cell migration in KYSE510; Figure S5: ChIP-qPCR showing p65 enrichment over the enhancer E2; Figure S6: p65 regulates the expression of SERPINB2 in KYSE510 cells; Table S1: Sequences of ASOs targeting SERPINB2; Table S2: Sequences of primers for qRT-PCR; Table S3: Quality control for RNA-seq samples; Table S4: Sequences of primers for ChIP PCR; Table S5: Differentially expressed gene list.

## Abbreviations

EGCG: epigallocatechin gallate; *SERPINB2*: Serine Proteinase Inhibitor 2; DEGs: differentially expressed genes; RELA: RELA Proto-Oncogene, NF-κB Subunit; ChIP: Chromatin Immunoprecipitation; SD: standard deviation.
