# Supplementary material for: SERPINB2, an Early Responsive Gene to Epigallocatechin Gallate, Inhibits Migration and Promotes Apoptosis in Esophageal Cancer Cells
Source: Cells. 2022 Nov 30;11(23):3852. doi: 10.3390/cells11233852 (PMC9738437; doi:10.3390/cells11233852)
Supplement: Supplementary file 1 [file cells-11-03852-s001.zip › cells-2025271-supplementary-Figures.pdf]

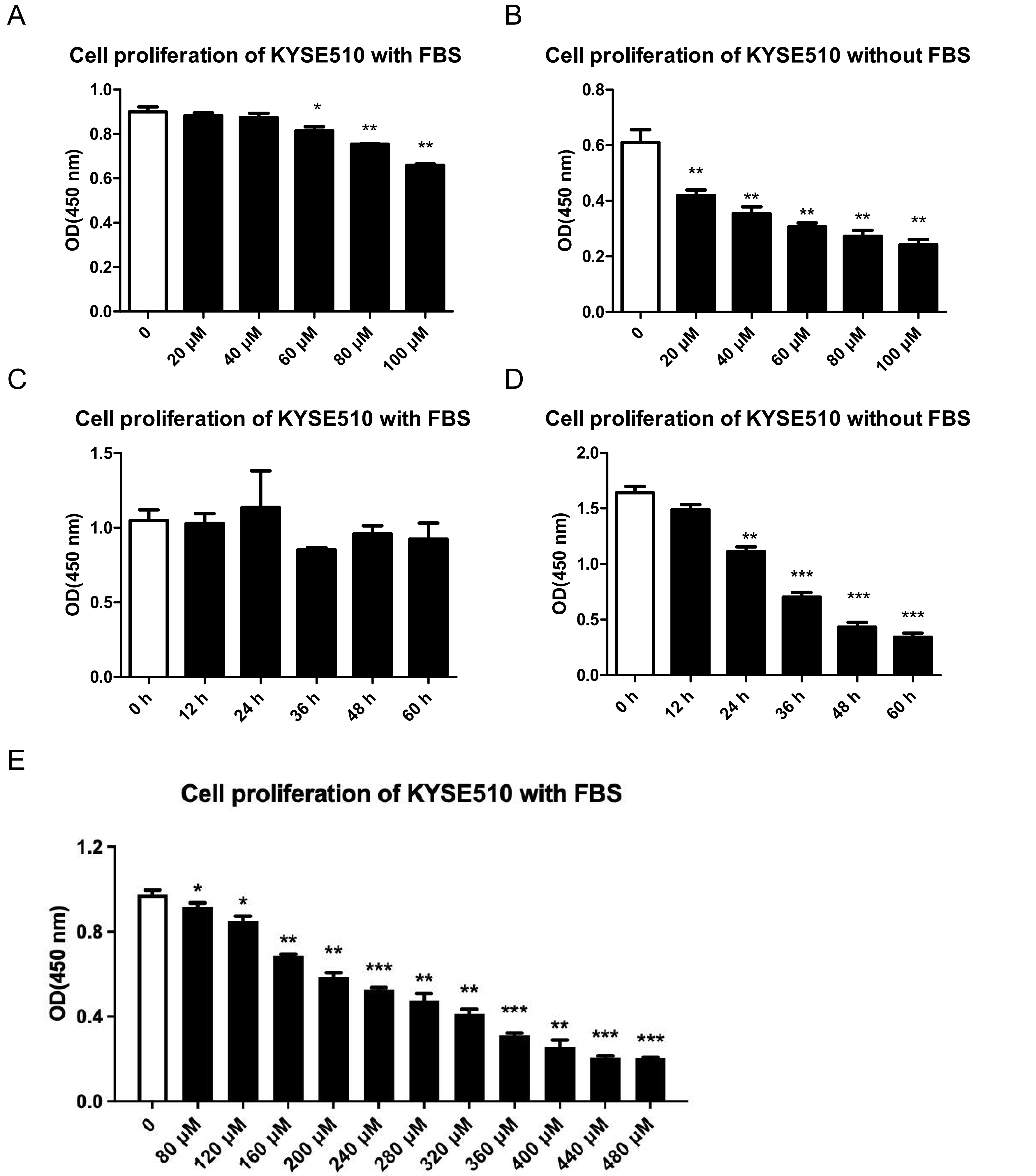

Supplementary Figure S1. Cell proliferation of KYSE510 under different conditions. A-B. Cells were treated with EGCG at concentrations from 0~100 μM with (A) or without (B) FBS for 24 h. C-D. Cells were treated with 60 μM EGCG at a series of time points from 0~60 h with (A) or without (B) FBS. E. Cells were treated with EGCG at relatively higher concentrations with FBS for 24 h. Data are shown as mean±SD. n=3, *: $P<0.05$; **: $P<0.01$; ***: $P<0.001$, ns: not significant.

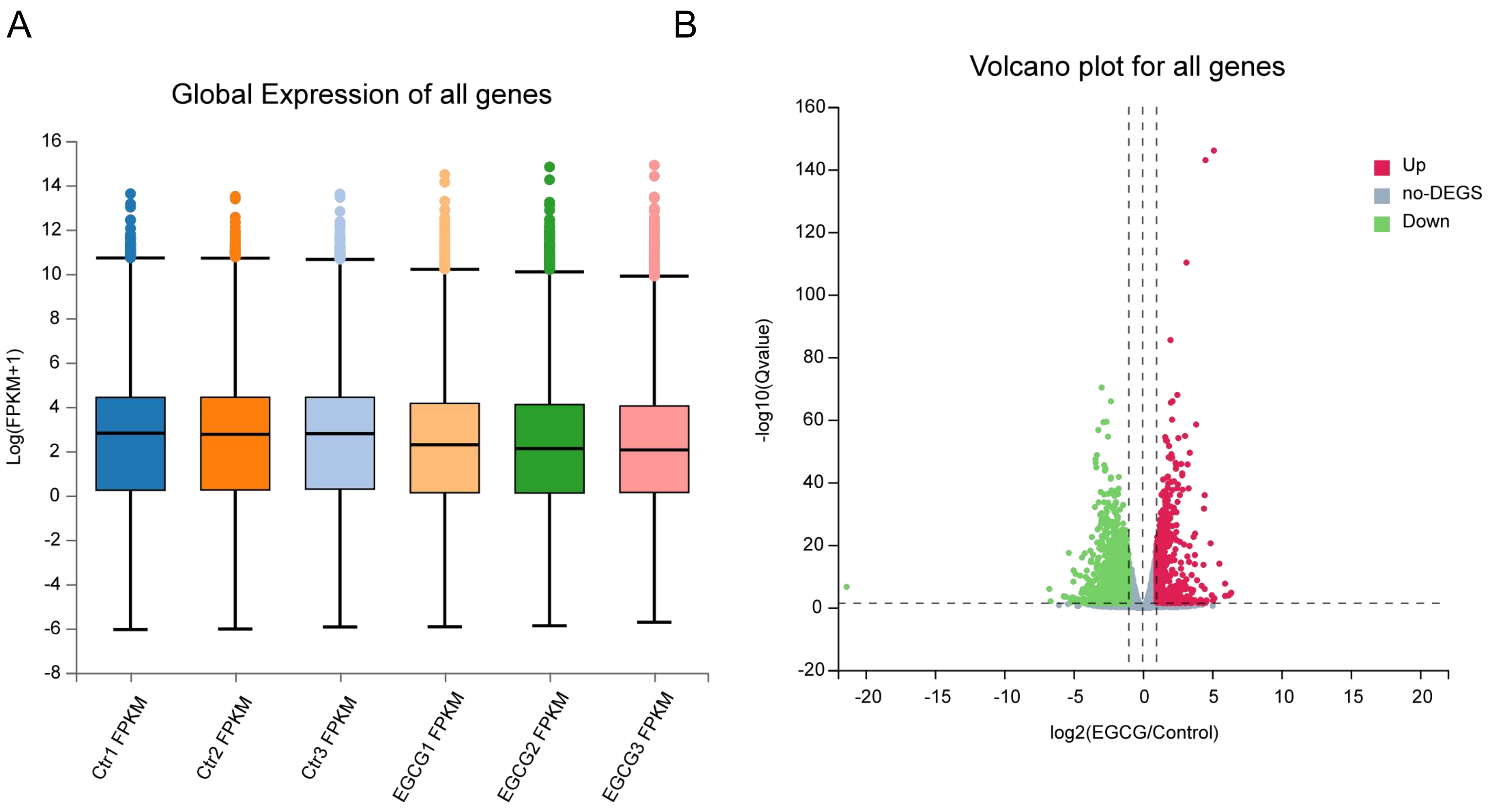

Supplementary Figure S2. Global analysis for RNA-seq of KYSE150 cells. A. The global expression of all detected genes in the control and EGCG-treated cells. B. Volcano plot showing the up-regulated, down-regulated, and non-regulated genes.

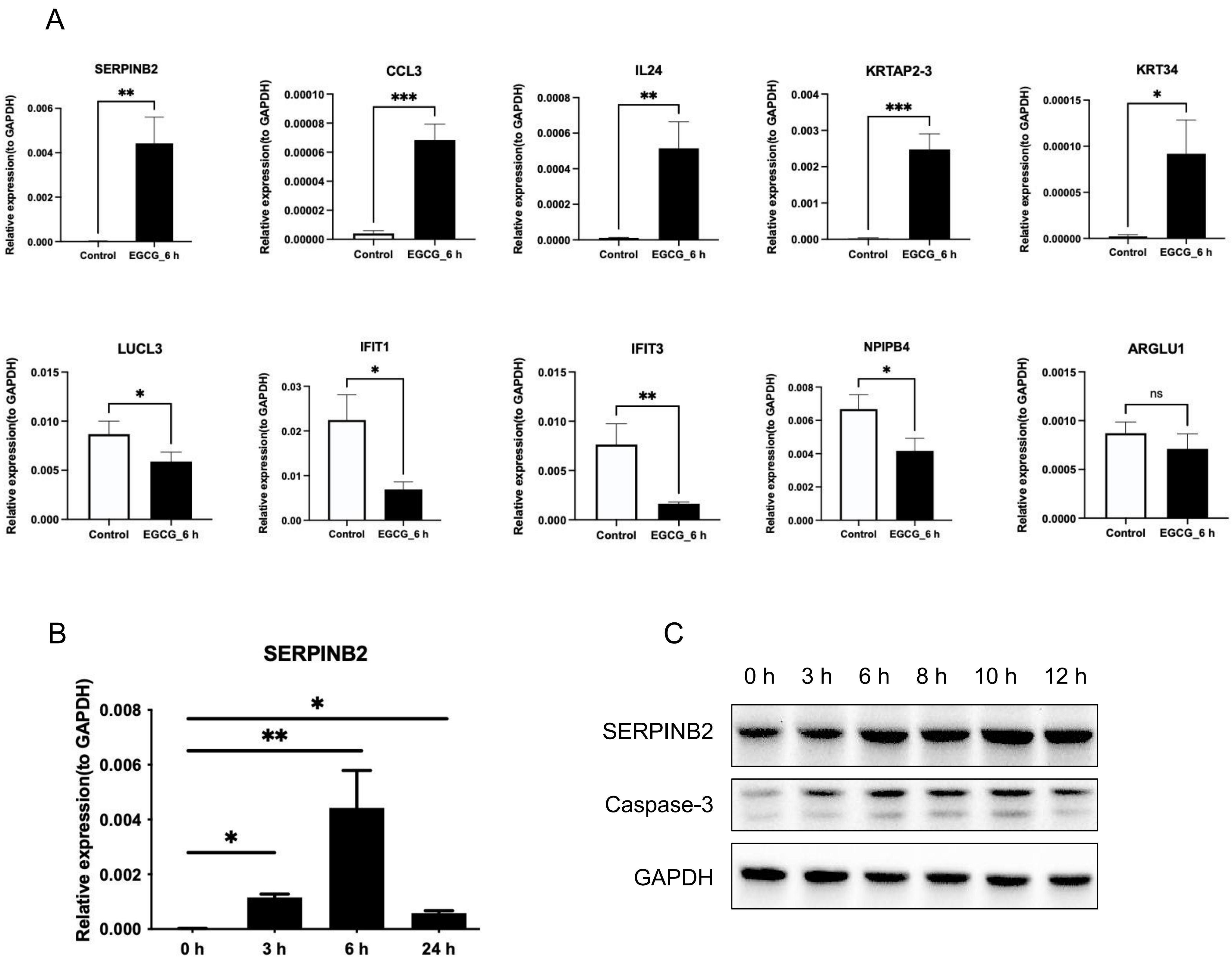

Supplementary Figure S3. RT-qPCR to verify DEGs in KYSE510 cells. A. Verification of top 10 up-regulated and down-regulated genes after EGCG treatment for 6 h in KYSE510 cells. B. Relative expression of SERPINB2 after EGCG treatment at 0, 3, 6, and 24 h in KYSE510 cells. C. Changes of SerpinB2 and Caspase-3 protein levels at different time points in KYSE510. Data are shown as mean ± SD. n=3, *: $P<0.05$; **: $P<0.01$; ***: $P<0.001$, ns: not significant.

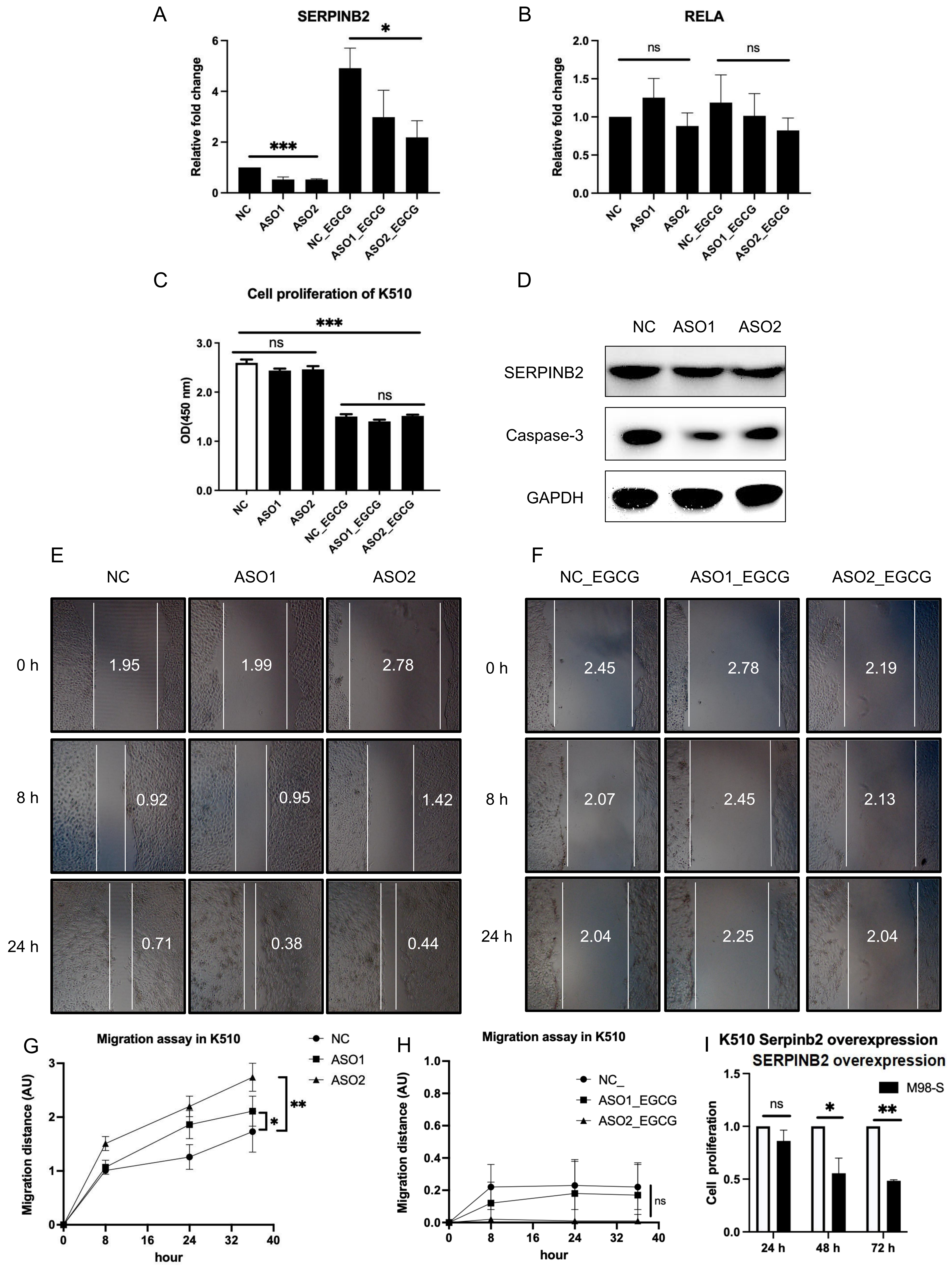
A
SERPINB2
Relative fold change
***
*
NC
ASO1
ASO2
NC_EGCG
ASO1_EGCG
ASO2_EGCG
B
RELA
ns
ns
C
Cell proliferation of K510
OD(450 nm)
D
SERPINB2
Caspase-3
GAPDH
E
F
0 h
8 h
24 h
1.95
1.99
2.78
0.92
0.95
1.42
0.71
0.38
0.44
2.45
2.78
2.19
2.07
2.45
2.13
2.04
2.25
2.04
G
Migration assay in K510
Migration distance (AU)
hour
**
H
NC_
ns
I
K510 Serpinb2 overexpression
SERPINB2 overexpression
M98-S
Cell proliferation
24 h
48 h
72 h

Supplementary Figure S4. SERPINB2 is associated with cell migration in KYSE510. A-B. Relative fold change of SERPINB2 (A) and RELA (B) 24 h after knockdown with ASO targeting SERPINB2. C. Cell proliferation after ASO-mediated knockdown of SERPINB2. D. Western blotting showing down-regulation of SerpinB2 and Caspase-3 after SERPINB2 knockdown. E-F. Wound healing assay showing cell migration after SERPINB2 knockdown without (E) or with (F) EGCG treatment. G-H. Statistical analysis for cell migration distance of three independent assays without (G) or with (H) EGCG. I. Cell proliferation after SERPINB2 overexpression in KYSE510. Data are shown as mean ± SD. n=3, *: $P<0.05$; **: $P<0.01$; ***: $P<0.001$, ns: not significant.

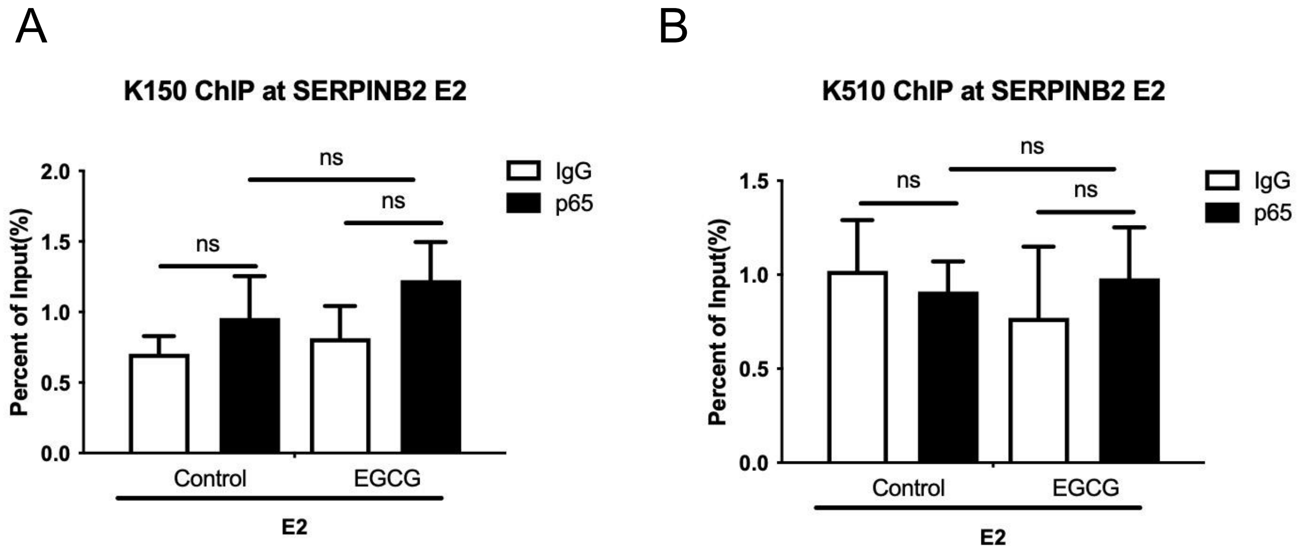

Supplementary Figure S5. ChIP-qPCR showing p65 enrichment over the enhancer E2. A. p65 enrichment in KYSE150 cells before and after EGCG treatment. B. p65 enrichment in KYSE510 cells before and after EGCG treatment. Data are shown as mean ±SD. n=3, ns: not significant.

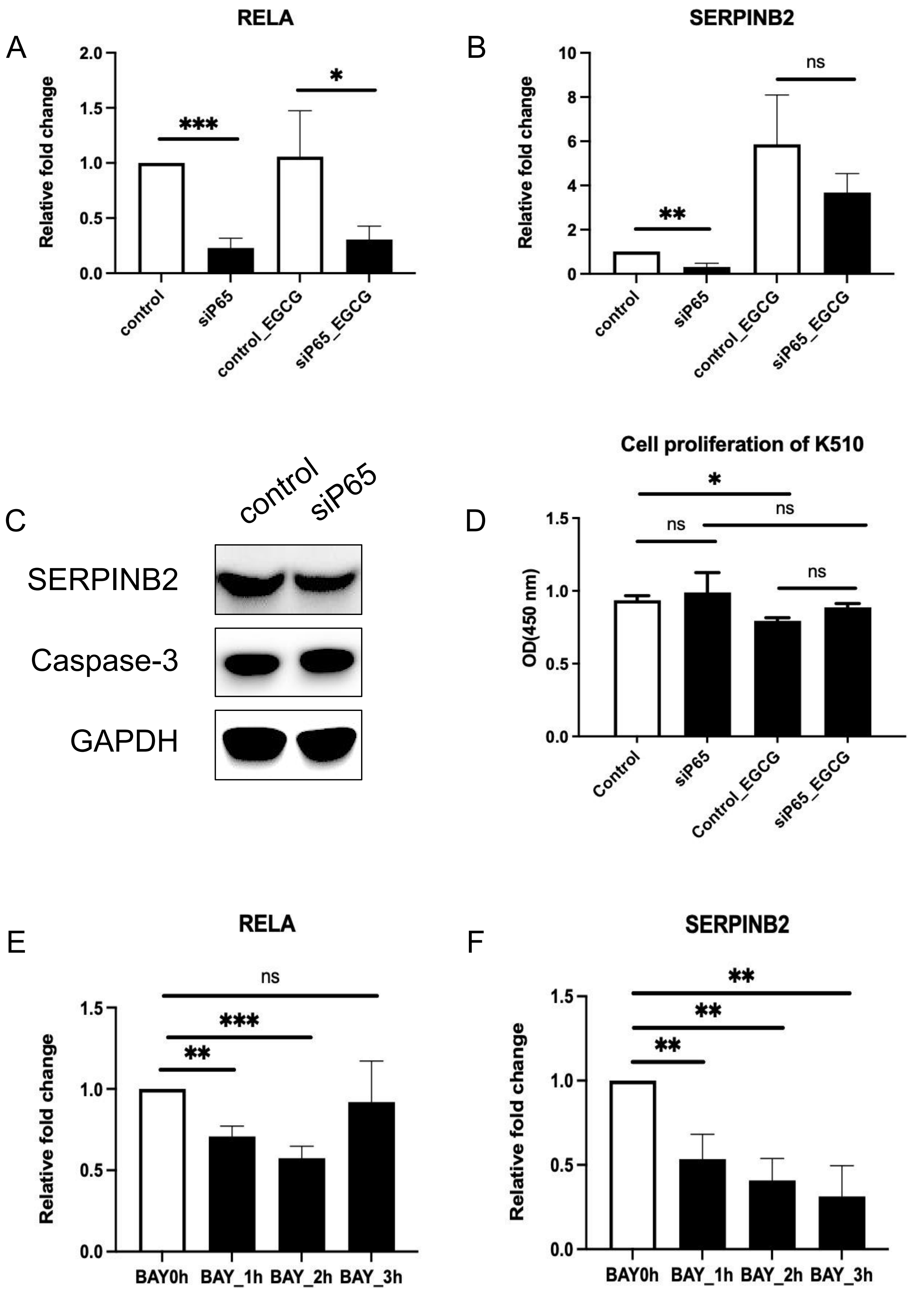

Supplementary Figure S6. p65 regulates the expression of SERPINB2 in KYSE510 cells. A-B. Relative fold change of RELA (A) and SERPINB2 (B) after p65 knockdown with or without EGCG. C. Western blotting showing protein levels of SerpinB2 and Caspase-3 after p65 knockdown. D. Cell proliferation after p65 knockdown. E-F. Relative fold change of RELA (E) and SERPINB2 (F) expression after p65 inhibition. Data are shown as mean ± SD. n=3, *: $P<0.05$; **: $P<0.01$; ***: $P<0.001$, ns: not significant.
